# Targeted liposomal nano-therapy combining anthocyanin and cisplatin reduces Ehrlich ascites carcinoma burden

**DOI:** 10.3389/fphar.2025.1659575

**Published:** 2025-10-03

**Authors:** Mai G. Awad, Sara H. El-Shafiey, Ramadan A. Ali, Dalia D. Abd El-Monem, Abdelnaser A. Badawy, Rasha Hamed Al-Serwi, Mohammed A. El-Magd, Nemany A. N. Hanafy

**Affiliations:** ^1^ Zoology Department, Faculty of Women for Arts, Science and Education, Ain Shams University, Cairo, Egypt; ^2^ Department of Biochemistry, Faculty of Medicine, Northern Border University, Arar, Saudi Arabia; ^3^ Department of Basic Dental Sciences, College of Dentistry, Princess Nourah bint Abdulrahman University, Riyadh, Saudi Arabia; ^4^ Department of Anatomy, Faculty of Veterinary Medicine, Kafrelsheikh University, Kafrelsheikh, Egypt; ^5^ Group of Bionanotechnology and Molecular Cell Biology, Institute of Nanoscience and Nanotechnology, Kafrelsheikh University, Kafrelsheikh, Egypt

**Keywords:** anthocyanin, liposome, cisplatin, Ehrlich ascites carcinoma, drug delivery systems, target therapy

## Abstract

**Background:**

Ehrlich ascites carcinoma (EAC), a rapidly growing tumor model, poses challenges in chemotherapy due to toxicity and resistance. Liposomal drug delivery improves anticancer therapy by enhancing bioavailability, targeting, and reducing systemic toxicity. Cisplatin (Cis), although effective, induces severe hepatic and renal toxicity. Anthocyanins (Ant), natural flavonoids with antioxidant and anticancer activities, can synergize with chemotherapy and reduce toxicity. This study evaluated three nano-liposomal formulations for EAC: Ant-loaded liposomes (Ant Ls), Cis-loaded liposomes (Cis Ls), and their combination (Cis + Ant Ls).

**Methods:**

Nano-liposomes containing Cis and/or Ant were prepared by thin-film hydration, functionalized with folic acid for targeting, and characterized by TEM for size and morphology. Encapsulation efficiency and drug release (48 h, dialysis) were determined. Cytotoxicity was tested on HCT 116 and Vero cells (MTT assay). Serum biochemical markers (ALT, AST, urea, and creatinine) were quantified, while tumor tissues were analyzed for apoptotic (caspase-3, Bcl2), inflammatory (IL1β), angiogenic (VEGF), metastatic (MMP9), and antioxidant (Nrf2, HO-1) genes using qPCR.

**Results:**

Ant Ls demonstrated a high encapsulation efficiency of 93.06% and exhibited sustained release profiles, achieving the highest cumulative drug release of 59.11% at 48 h. Cis Ls and/or Ant Ls demonstrated significant cytotoxic (P < 0.05) effects on HCT 116 colon cancer cells while exhibiting minimal toxicity to normal Vero cells. In comparison to untreated EAC controls, mice treated with Cis Ls and/or Ant Ls exhibited significantly (P < 0.05) enhanced liver and renal function and structure, as evidenced by reductions in ALT, AST, urea, and creatinine and histopathology lesions. The treatments also decreased EAC burden as noticed by a reduction in total and viable tumor cell counts, and ascitic fluid volume, along with an increase in non-viable cells. The anticancer effect of Cis Ls and/or Ant Ls was attributed to multiple mechanisms, including increased apoptosis, reduced inflammation, inhibited angiogenesis, and suppression of metastasis. The combined treatment enhanced antioxidant defenses to mitigate Cis toxicity. The combination of Cis Ls and Ant Ls markedly decreased tumor burden and demonstrated therapeutic synergy, suggesting potential for improved outcomes.

**Conclusion:**

Cis Ls and Ant Ls delivery strategy mitigates liver and kidney damage while enhancing anticancer efficacy against EAC.

## Graphical Abstract

**Figure:**
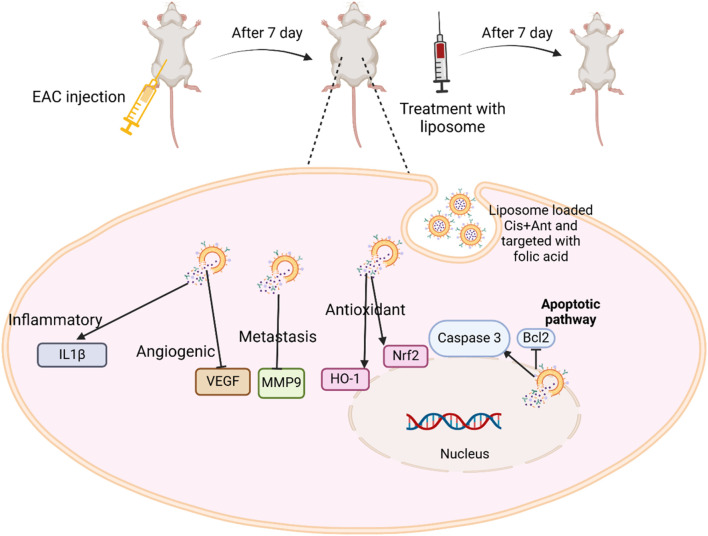
The experimental process and molecular mechanisms involved the Cis + Ant Ls treatment in EAC cells.

## 1 Introduction

Cancer remains one of the leading causes of mortality worldwide, necessitating innovative therapeutic strategies that can enhance efficacy while minimizing adverse effects (Hanahan and Weinberg, 2011). Ehrlich ascites carcinoma (EAC), a rapidly proliferating transplantable tumor, is widely used as an experimental model to evaluate anticancer therapies due to its aggressive nature and ease of propagation (Magdy et al., 2020; Tawfic et al., 2024a; Tawfic et al., 2024b). EAC is a transplantable, undifferentiated tumor derived from a spontaneous murine mammary adenocarcinoma. It is widely used in preclinical cancer research due to its rapid proliferation, high transplant ability, and predictable tumor progression in mice (Elbialy and Mady, 2015). Compared to chemically induced or genetically engineered models, which require prolonged induction periods, high costs, and often display inter-animal variability, EAC provides a more consistent, time-efficient, and cost-effective platform for evaluating anticancer agents (Magdy et al., 2020; Tawfic et al., 2024b). The intraperitoneal growth of EAC also facilitates direct assessment of tumor volume and therapeutic response via ascitic fluid analysis. Nonetheless, its limitations include the lack of a tissue-specific tumor microenvironment and limited capacity to model metastatic spread or heterogeneity seen in human solid tumors. Despite this, EAC remains a valuable model for screening cytotoxic drugs and studying tumor-host interactions *in vivo*.

Among the promising delivery systems being explored, liposomes (Ls) have attracted considerable attention in oncology for their biocompatibility, ability to encapsulate both hydrophilic and hydrophobic drugs, and potential to improve the therapeutic index of chemotherapeutics (Hamad et al., 2024; Nsairat et al., 2022). By enhancing bioavailability and enabling targeted delivery, Ls can minimize systemic toxicity and improve drug accumulation at tumor sites (Chen et al., 2024). Specifically, liposomal delivery has shown encouraging results in targeting EAC cells, making it a valuable platform for investigating novel treatment combinations (Elbialy and Mady, 2015).

Cisplatin (Cis), a prevalent chemotherapeutic drug, targets diverse malignancies by generating DNA damage and death. Nonetheless, its therapeutic use is often constrained because of significant adverse effects, including nephrotoxicity, neurotoxicity, and ototoxicity, in addition to the development of drug resistance (Abass et al., 2024; Awad et al., 2020). Cis Ls formulations are designed to improve the targeting of neoplastic cells and minimize their effects on healthy organs. Liposomal administration enhances the bioavailability of Cis at the tumor site, ensuring higher dosages are administered to cancer cells, and enhances therapeutic effectiveness in EAC treatment (Ledezma-Gallegos et al., 2020).

Anthocyanins (Ants), natural polyphenolic chemicals present in several fruits and vegetables (including red beetroot), have significant antioxidant, anti-inflammatory, and anticancer activities, making them intriguing supplements to traditional chemotherapy (Wang and Stoner, 2008). As they are sourced from natural origins, Ants provide a therapeutic option with fewer adverse effects than traditional chemotherapy (Gaikwad et al., 2023). Research suggests that Ants can help mitigate Cis-induced toxicity by reducing oxidative stress and inflammation, thereby protecting normal cells (Awad et al., 2024a; Awad et al., 2024b). Additionally, Ants have been shown to enhance Cis’s anticancer efficacy by modulating key signaling pathways such as PI3K/Akt, NF-κB, and MAPK, leading to increased apoptosis and tumor suppression (Kowalczyk et al., 2024; Paramanantham et al., 2020). Cis efficiently eradicates cancer cells by inducing DNA damage, whilst Ant amplifies this impact via its antioxidative and pro-apoptotic characteristics (Cheng et al., 2018; Renault-Mahieux et al., 2021). The synergistic effect of Ants in scavenging reactive oxygen species (ROS) may alleviate oxidative damage induced by Cis, concurrently augmenting cancer cell apoptosis (Awad et al., 2024a; Awad et al., 2024b). However, Ants therapeutic potentials face limitations due to insufficient bioavailability, fast disintegration, and suboptimal absorption (Karthikeyan et al., 2020; Shen et al., 2022). So, Ant Ls formulations seek to address these limitations by encapsulating Ants in liposomes, improving their stability and bioavailability (Lin et al., 2017).

The synergistic antitumor effects and capacity to combat multidrug resistance are major reasons for the increased interest in the co-administration of diverse drugs or a mix of chemotherapy and natural products (Attia et al., 2022; Awad et al., 2020; Badawy et al., 2021; Selim et al., 2019). Compared to the results of individual treatments, the combination treatment was often more effective in cytotoxic, apoptotic, antioxidant, anti-inflammatory, anti-angiogenic, and anti-metastatic measures. The results highlight the possibility of improving anticancer effects by combining Cis + Ant Ls. Every treatment component has special advantages; Cis is a well-known chemotherapeutic drug; Ants have anti-inflammatory and antioxidant effects. These results suggest that by concurrently targeting various pathways linked to cancer, the Cis + Ant Ls therapy could offer a full strategy for inhibiting the growth and dissemination of malignancies. This combo treatment can reduce the required dosage of Cis, lowering the medication’s harmful side effects by using ants’ natural features to improve anticancer activity.

While Cis Ls (Ledezma-Gallegos et al., 2020) and Ant Ls (Lin et al., 2017) have been investigated for targeted therapy, their combined liposomal formulation has not been evaluated for anticancer efficacy. Combining Cis and Ant in one capsule would offer a potential approach for treating EAC. Employing liposomal carriers for both Cis and Ant facilitates concurrent delivery to cancer cells, enhances treatment effectiveness, and mitigates the systemic toxicity often associated with free Cis. Therefore, this study aimed to evaluate the therapeutic efficacy of this combined approach in EAC.

## 2 Materials and methods

### 2.1 Chemicals and reagents

HCT 116 human colorectal carcinoma cells and Vero (African green monkey kidney epithelial) cells were obtained from the National Cancer Institute in Cairo, Egypt. Cis (with the commercial name CISPLATINE MYLAN; Code DCI: 05G111), chitosan, and folic acid (FA) were purchased from Sigma-Aldrich (St. Louis, MO, United States). Tissue culture reagents [DMEM 4.5 g/L Glucose with L-Glutamine, fetal bovine serum (FBS), penicillin/streptomycin, trypsin, dimethyl sulfoxide (DMSO), MTT, Trypsin-EDTA 10x solution] were supplied by Sigma and GIBCO (New York, United States), and Lonza Bioproducts (Verviers, Belgium). Gene JET RNA Purification Kit was obtained from Thermo Scientific (Waltham, MA, United States), while the Quantiscript Reverse Transcription Kit and Quanti-Tect SYBR Green PCR Kit were purchased from Qiagen (Hilden, Germany). Soybean phospholipids were purchased from Avanti Polar Lipids (Alabaster, AL, United States). Primers were synthesized by Integrated DNA Technologies (Coralville, IA, United States). All other chemicals and reagents were of the highest purity available.

### 2.2 Extraction of Ant

Ant was extracted by homogenizing an equal ratio (1/1 w/v) of sample (200 g peeled beetroot fruit) and solvent (200 mL 95% ethanol) for 2 h at 65 ˚C. After the color had changed to dark red, the extract was filtered and evaporated to dryness from ethanol in an oven at 40 °C for 48 h, washed, and dissolved in distilled water, then kept in a dark bottle at 4 °C until used for liposome fabrication (Awad et al., 2024b).

### 2.3 Distinguishing between Ant and betacyanin

Under acidic conditions, Ant and betacyanin exhibited distinct behaviors that help differentiate them. When treated with hot hydrochloric acid (HCl), Ant remained stable and retained their original color, whereas betacyanin degraded and lost their pigmentation. In contrast, under alkaline conditions (with sodium hydroxide, NaOH), Ant shifted to a blue-green hue that might fade over time, while betacyanin transformed into a stable yellow color. These contrasting responses to pH changes were a useful diagnostic tool for identifying these pigments in plant extracts (Nielson and Harley, 2010).

### 2.4 Quantification of total Ant

The total Ant content of beetroot extract was determined using the pH differential method (Jumaa Jandal, 2025). This method relies on the property of Ant to alter their color in response to changes in the pH of the solution. At an acidic pH of approximately 1, the substances appeared red; conversely, at neutral or slightly basic pH levels around 4.5, they exhibited purple or blue. To determine the total Ant content, we measured the absorbance of the extract at two wavelengths (520 nm and 700 nm) and two pH levels (1 and 4.5). Total Ant content (mg/L) was calculated as using this formula: $\frac{\left[\left(A520-A700\right)\text{pH}1-\left(A520-A700\right)\text{pH}4\;\right]\times \text{MW}\times \text{DF}\times 1000}{\epsilon \times L}$ , where A520 and A700 represent the absorbance values at 520 nm and 700 nm; MW denotes the molecular weight of cyanidin-3-glucoside (which is 449.2 g/mol and serves as a reference compound); DF indicates the dilution factor (which was calculated as 25 divided by 0.5, resulting in a value of 50); ϵ refers to the molar absorptivity of cyanidin-3-glucoside (quantified at 26,900 L/mol/cm); and L signifies the path length of the cuvette, typically 1 cm (Fierri et al., 2023).

### 2.5 Liposomal fabrication of Ant and/or Cis

The liposomal formulation of Ant, Cis, and their combination (Cis + Ant) was synthesized via a modified thin-film hydration method as previously detailed (Abd El- Salam et al., 2023; Bahrami Parsa et al., 2023). The lipid phase was created in each formulation by dissolving 20 mg of soybean phospholipid in 0.5 mL of chloroform and 3 mL of ethanol while magnetically stirring at 70 °C (600 rpm for 5 min). Subsequently, 20 mg of cholesterol, similarly dissolved in the same solvent combination, was integrated. Hydration was accomplished using 40 mL of distilled water, and surface modification included the addition of 5 mg of folic acid mixed with 20 mL of albumin (50 mg/100 mL). The suspensions were sonicated for 15 min and then dialyzed against distilled water for 24 h before lyophilization and storage at −20 °C. For Ant Ls, 100 mg of Ant was first sonicated in 2 mL (25 mg/100 mL) chitosan before adding the lipid phase. Cis Ls were generated by sonicating 2 mg of Cis in 200 µL of polygalacturonic acid (50 mg/mL) and 1 mL of chitosan (25 mg/100 mL) before their integration into the lipid mixture. The Cis + Ant liposomes were generated by integrating both drug solutions (Ant in chitosan and Cis in polygalacturonic acid/chitosan) into the lipid phase before hydration. This standardized and versatile method facilitated the effective encapsulation of one or two pharmaceuticals into a singular liposomal delivery system.

### 2.6 Characterization of liposomes

The prepared liposomes were characterized using transmission electron microscopy (TEM) (Inkson, 2016) and Fourier transform infrared (FTIR) spectroscopy (Mahamuni and Adewuyi, 2009). For TEM analysis, 1 mL of each diluted sample was sonicated for 5 min to ensure homogeneity. The sample was then applied to carbon-coated copper grids, air-dried for 15 min, and negatively stained with phosphotungstic acid. Imaging was performed using a 200 kV JEOL JEM 2100F TEM. For FTIR analysis, surface molecular structures were assessed in the 500–4,000 cm^−1^ range via the KBr pellet method using a JASCO FTIR spectrometer (Model AUP1200, Japan). Each sample was scanned at least three times across different regions, and representative spectra were analyzed for functional group identification.

### 2.7 Determination of encapsulation efficiency (EE)

The following equation was used: EE (%) = $\frac{\text{Total}\;\text{amount}\;\text{of}\;\text{Ant}\;\left(\text{mg}\right)-\text{Amount}\;\text{of}\;\text{Ant}\;\text{in}\;\text{the}\;\text{supernatant}}{\text{Total}\;\text{amount}\;\text{of}\;\text{Ant}\;}\times 100$ (Amararathna et al., 2022) to determine the concentration of Ant within the capsule. The total Ant in the extract and supernatant was obtained following the centrifugation of the Ant Ls. The total amount of Ant and the quantity in the supernatant after encapsulation were assessed using the pH differential technique.

### 2.8 *In vitro* release of Ant Ls and/or Cis Ls

The *in vitro* release of entrapped Ant and Cis from liposomes was checked utilizing the dialysis bag diffusion method (Gupta and Trivedi, 2018). The dialysis tubing was soaked in distilled water overnight to facilitate the complete expansion of the dialysis membrane prior to cutting it into suitable segments. Liposomes containing Ant, Cis, or Cis + Ant (5 mL) were each placed into a sealed membrane dialysis bag. A dialysis bag was immersed in 200 mL of phosphate-buffered saline solution at pH 7.4. Ethanol was incorporated into the medium to ensure the establishment of sink conditions. The system was maintained at 37 ± 0.5 °C with continuous magnetic stirring at 400 rpm. Glass beakers were sealed with Parafilm to prevent the volatilization of alcohol (Yan et al., 2010). To ensure a constant volume of the receptor medium, 3 mL aliquots were removed at designated intervals and replaced with an equivalent volume of fresh media. The collected samples underwent analysis via UV spectroscopy.

### 2.9 Evaluation of cell viability by MTT assay

Cell viability was assessed with the MTT assay, which evaluates mitochondrial reductase activity in viable cells by converting tetrazolium salts into formazan crystals. Cells were inoculated at a density of 10,000 cells per well in full growth media (DMEM supplemented with 10% FBS and 1% antibiotics) and allowed to adhere for 24 h. Upon reaching 70%–80% confluence, cells were subjected to successive dilutions (0–100 μg/mL) of Cis Ls and/or Ant Ls for a duration of 24 h. After treatment, MTT reagent (5 mg/mL) was introduced, and cells were incubated for 4 h to facilitate formazan crystal formation. The crystals were dissolved in DMSO, and absorbance was recorded at 570 nm using a microplate reader. The half-maximal inhibitory concentration (IC_50_) was determined using dose-response curves produced with GraphPad Prism software. Cell viability percentages were standardized to untreated controls (deemed 100% viable) using the formula: $\frac{\left(\text{OD}\;\text{treated}-\text{OD}\;\text{blank}\right)}{\left(\text{OD}\;\text{control}-\text{OD}\;\text{blank}\right)\;}\times 100$ .

### 2.10 Animals and experimental design

All procedures involving animals followed ARRIVE guidelines and were ethically approved by the Animal Ethics Committee at the Faculty of Women for Arts, Science, and Education, Ain Shams University (Approval Code: ASU/W/Sci-5R/23-1-16). Female Swiss albino mice (10–12-week-old and weighing 20–25 g) were housed under a regulated temperature in a 12-12-h light-dark cycle. Before the trial began, each mouse spent 2 weeks acclimating. The animals ate the same food and had full water access. Thirty-five mice were divided into five groups (n = 7 for each group): normal control (Cnt), EAC, Cis Ls, Ant Ls, and Cis + Ant Ls. The Cnt group was given an intraperitoneal (i.p) injection of normal saline (0.9% w/v, 200 μL/mouse). The EAC group was i. p injected with 200 µL containing 1 × 10^6^ EAC cells and received no therapeutic intervention. EAC cells were sourced from three EAC-bearing mice obtained from the National Institute of Oncology at Cairo University, Egypt, after 14 days of intraperitoneal injection. These mice in our lab underwent repeated aseptic intraperitoneal transplanting using the ascitic fluid containing EAC cells. In the Cis Ls group, EAC was induced as in the previous group, but animals were i. p injected with 200 μL (50 mg/kg) Cis Ls for seven consecutive days starting from Day (D) 8 to D14 post-EAC injection (Franco et al., 2021). The Ant Ls group was manipulated as the Cis Ls group, but mice were i. p injected with 200 μL (100 mg/kg) Ant Ls (Carvalho et al., 2017). The Cis + Ant Ls group received an i. p injection of 200 µL containing 1 × 10^6^ EAC cells at D1 and was i. p injected with 50 mg/kg Ant Ls and 50 mg/kg Cis Ls over seven consecutive days from D8 to D14. After the experimental period (2 weeks), mice were euthanized 24 h following the final treatment, and blood samples were subjected to centrifugation in sterile glass tubes for 15 min at 3,000×g to obtain clear, non-hemolyzed sera. Eppendorf tubes labeled accordingly were promptly shipped to −20 °C, and the sera were frozen for subsequent biochemical analysis. Peritoneal fluids containing EAC cells were collected using a sterile syringe for cell counting, viability assessment, RNA extraction, and cellular pathology analysis. Livers and kidneys were promptly excised, and specimens were fixed in 10% formalin for pathological examination.

### 2.11 Ascitic fluid volume, cell count, and viability

After euthanasia, the peritoneal cavity was carefully opened, and the ascitic fluid was collected using a sterile syringe. The volume of ascitic fluid was measured directly using a graduated 5 mL syringe and recorded in milliliters for each mouse. EAC cells were counted using a hemocytometer following staining with 0.5% trypan blue. A trypan blue exclusion assay was performed as previously described (Strober, 2015). The viability percentage was calculated as follows: $\frac{\left(\text{total}\;\text{number}\;\text{of}\;\text{cells}-\text{number}\;\text{of}\;\text{trypan}\;\text{blue}\;\left(\text{dead}\right)\;\text{cells}\right)}{\text{total}\;\text{number}\;\text{of}\;\text{cells}}\times 100$ . (El-Magd et al., 2017).

### 2.12 Real-time PCR

Real-time PCR was used to assess alterations in the relative expression of the caspase3, *Bcl2*, *IL1β*, *VEGF*, *MMP9*, *Nrf2*, and *HO-1* genes in EAC cells across all groups. Total RNA was first extracted and then reverse-transcribed into cDNA using kits obtained from Thermo Scientific, United States (#K0731 and #EP0451, respectively). Table 1 lists the primer sequences. The 2^−ΔΔCT^ approach was used to evaluate the change in gene expression.

**TABLE 1:** Forward and reverse primer sequences for candidate mice genes.

Gene	Forward primer (^/^5 ------ ^/^3)	Reverse primer (^/^5 ------ ^/^3)
Caspase3	GAC​CAT​ACA​TGG​GAG​CAA​GT	CCT​TCA​TCA​CCA​TGG​CTT​AGA
Bcl2	CAT​GCC​AAG​AGG​GAA​ACA​CCA​GAA	GTG​CTT​TGC​ATT​CTT​GGA​TGA​GGG
IL1β	AAA​TCT​CGC​AGC​AGC​ACA​TCA​A	CCA​CGG​GAA​AGA​CAC​AGG​TAG​C
VEGF	GAT​CAT​GCG​GAT​CAA​ACCTCACC	CCT​CCG​GAC​CCA​AAG​TGC​TC
MMP9	TCG​AAG​GCG​ACC​TCA​AGT​G	TTC​GGT​GTA​GCT​TTG​GAT​CCA
Nrf2	CGA​GAT​ATA​CGC​AGG​AGA​GGT​AAG​A	GCT​CGA​CAA​TGT​TCT​CCA​GCT​T
HO-1	GCC​CAC​GCA​TAT​ACC​CGC​TAC​CT	CCA​TGG​CCT​TCT​GTG​CAA​TCT​TCT
GAPDH	TGG​CAA​AGT​GGA​GAT​TGT​TGC​C	AAG​ATG​GTG​ATG​GGC​TTC​CCG

### 2.13 Liver and kidney function measurement

Assess hepatic function in female mice by measuring aspartate aminotransferase (AST) and alanine aminotransferase (ALT). Creatinine in serum was measured using an alkaline picrate colorimetric method. The urea levels were estimated calorimetrically by measuring the amount of urea nitrogen in the serum using a commercially available kit (Diamond Diagnostics).

### 2.14 Cellular and tissue pathology

Following ascitic fluid collection, the samples were fixed with ethanol. Cells were dried on a microscope slide before staining with hematoxylin and eosin (H&E). Liver and kidney tissues undergo dehydration using a sequential series of ethanol alcohol concentrations. The specimen was subsequently cleared in xylene, embedded in paraffin wax, sectioned to a thickness of 5 μm, and stained by H&E. All specimens were imaged using a light microscope.

### 2.15 Statistical analysis

The statistical analysis was conducted using one-way ANOVA with GraphPad Prism 8 (GraphPad Software, Inc., La Jolla, CA, United States), followed by Tukey’s Honestly Significant Difference (Tukey’s HSD) test as a *post hoc* test. Data were presented as mean ± standard error of the mean (SEM). The significance was defined as P < 0.05.

## 3 Results

### 3.1 Characterization of Ant Ls and/or Cis Ls

**FIGURE 1:**
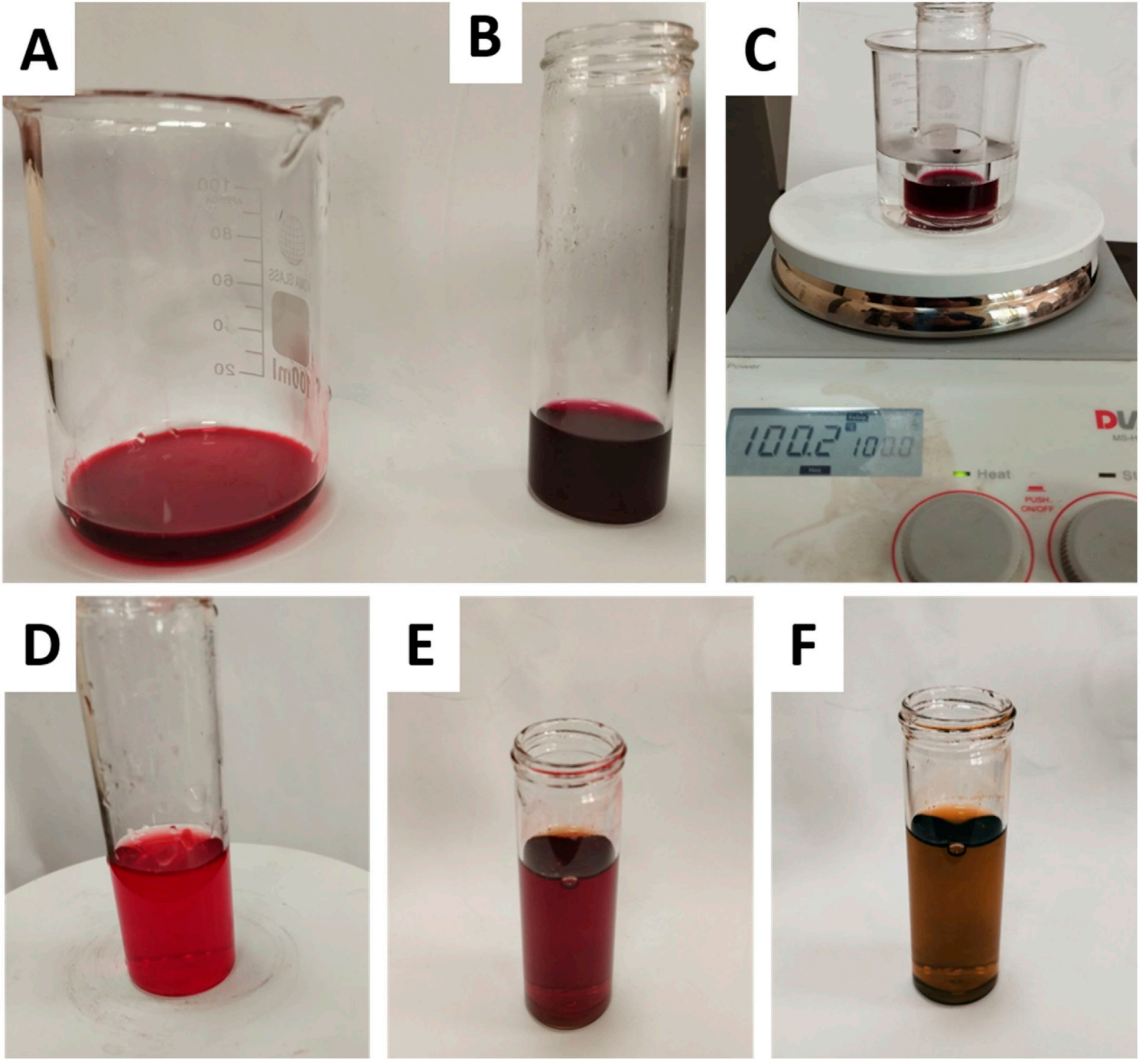
Colorimetric analysis of plant extract pigments under acidic and alkaline conditions. **(A)** untreated extract, **(B)** extract + HCl (acidic conditions), **(C)** extract + HCl after heating for 5 min (stability test under acidic heat), **(D)** untreated extract, **(E)** extract after dropwise addition of NaOH (transition to alkaline pH), and **(F)** extract after full addition of 3 mL NaOH (final alkaline conditions).

The colorimetric responses seen under different pH settings validate the existence of Ant in the extract. Following the addition of HCL and a 5-min heating period (Figures 1A–C), the pigments maintained their distinctive red coloration, indicating durability in the presence of hot HCL—a defining property of Ant. Conversely, under alkaline circumstances (Figures 1D–F), the pigments transitioned to a blue-green hue, aligning with the established behavior of Ant in sodium hydroxide, whereby their chromophores experience structural alterations resulting in bathochromic shifts. The lack of pigment degradation in acid or a yellow transition in alkali excludes the existence of betacyanin, which would destabilize acidic environments and maintain a stable yellow hue in basic circumstances. Consequently, the results designate the pigments as Ant.

**FIGURE 2:**
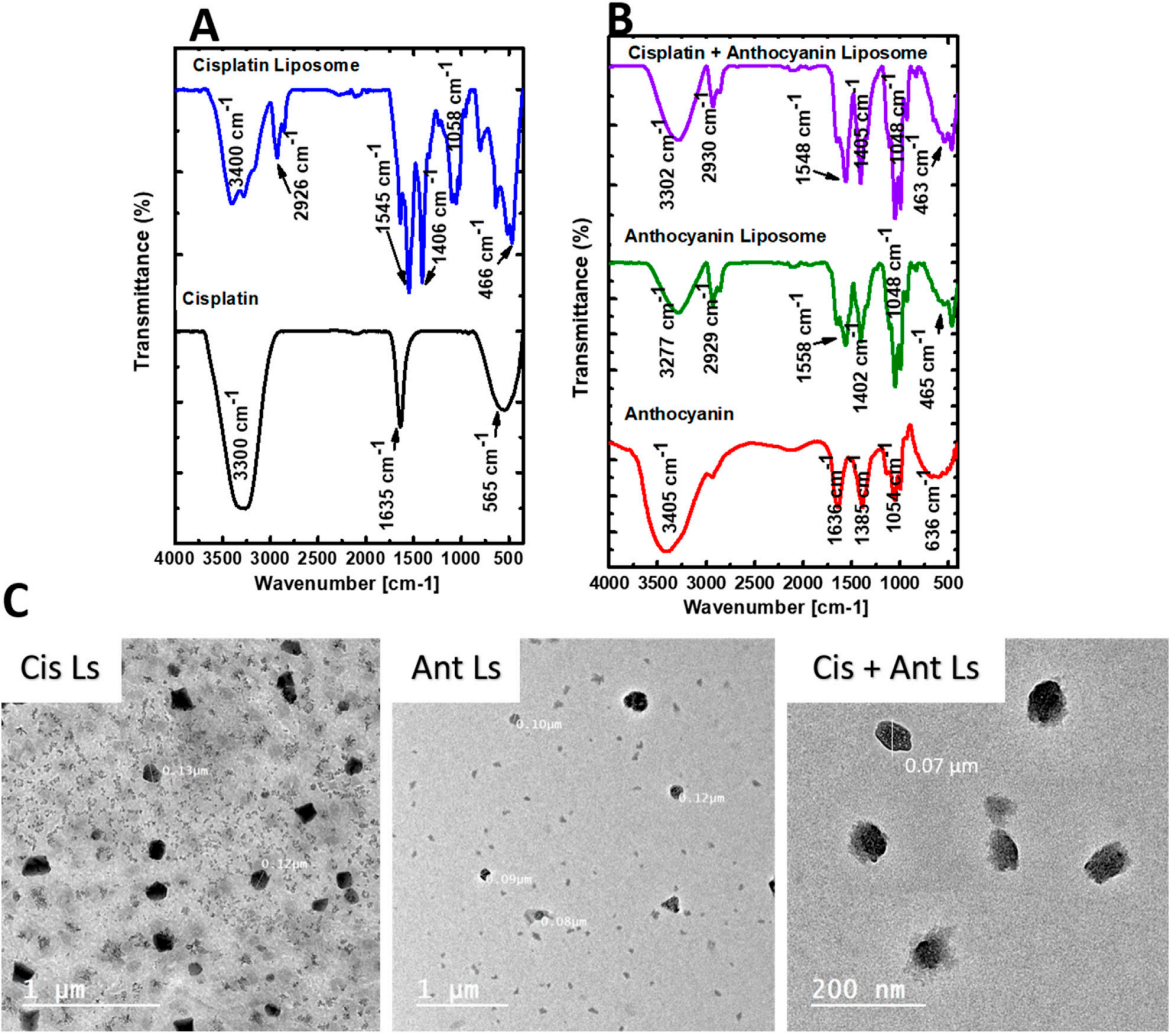
Characterization of Ant, Cis, and their Ls by FT-IR spectra and Transmission electron microscopy (TEM). **(A)** FT-IR spectra of Cis and Cis Ls. **(B)** FT-IR spectra of the Ant extract, Ant Ls, and Cis + Ant Ls. **(C)** TEM analysis reveals that the synthesized liposome nanoparticles (Cis Ls, Ant Ls, and Cis + Ant Ls) exhibit a size range of 70–130 nm.

The chemical structure of Cis was found to be intact inside Ls, according to the FTIR data (Figure 2A). The Cis and Cis Ls exhibited platinum ammonium bonds at 565 cm^−1^ and 466 cm^−1^, respectively. Tensional tremors of ammonium groups could be noticed at 3,300 cm^−1^ (Cis) and 3,400 cm^−1^ (Cis Ls) (Awad et al., 2024b; Babaei et al., 2019). The Ant and Ant Ls FT-IR spectra were recorded at a resolution of 0.1 cm^−1^ in the 4,000 cm^−1^ to 400 cm^−1^ range (Figure 2B). Ant Ls at 3,277 cm^−1^ also showed -OH stretching vibrations, which were thought to be responsible for the noticeable broadband at 3,266 cm^−1^ in the FT-IR spectra of Ant. Ant and Ant Ls showed the C=N stretching band at 1,645 cm^−1^ and 1,558 cm^−1^. In contrast to the strong presence in Ant Ls at 1,048 cm^−1^, the last faint band in Ant at 1,013 cm^−1^ was thought to be caused by C-H bending (Jeyaram and Geethakrishnan, 2020). It was at 3,302 cm^−1^ when the stretching vibrations of -OH were detected in the Cis + Ant Ls spectra. Ant and Cis were suggested to be present by the band at 1,405 cm^−1^. In contrast, Cis was indicated by the band at 1,548 cm^−1^ (Figure 2B). TEM analysis revealed that Ls were spherical and exhibited a consistent, uniform size distribution (Figure 2C). The diameters of the Ls ranged from 70 to 130 nm, confirming their structural homogeneity and suitability for experimental applications.

### 3.2 Quantification of total Ant content

**FIGURE 3:**
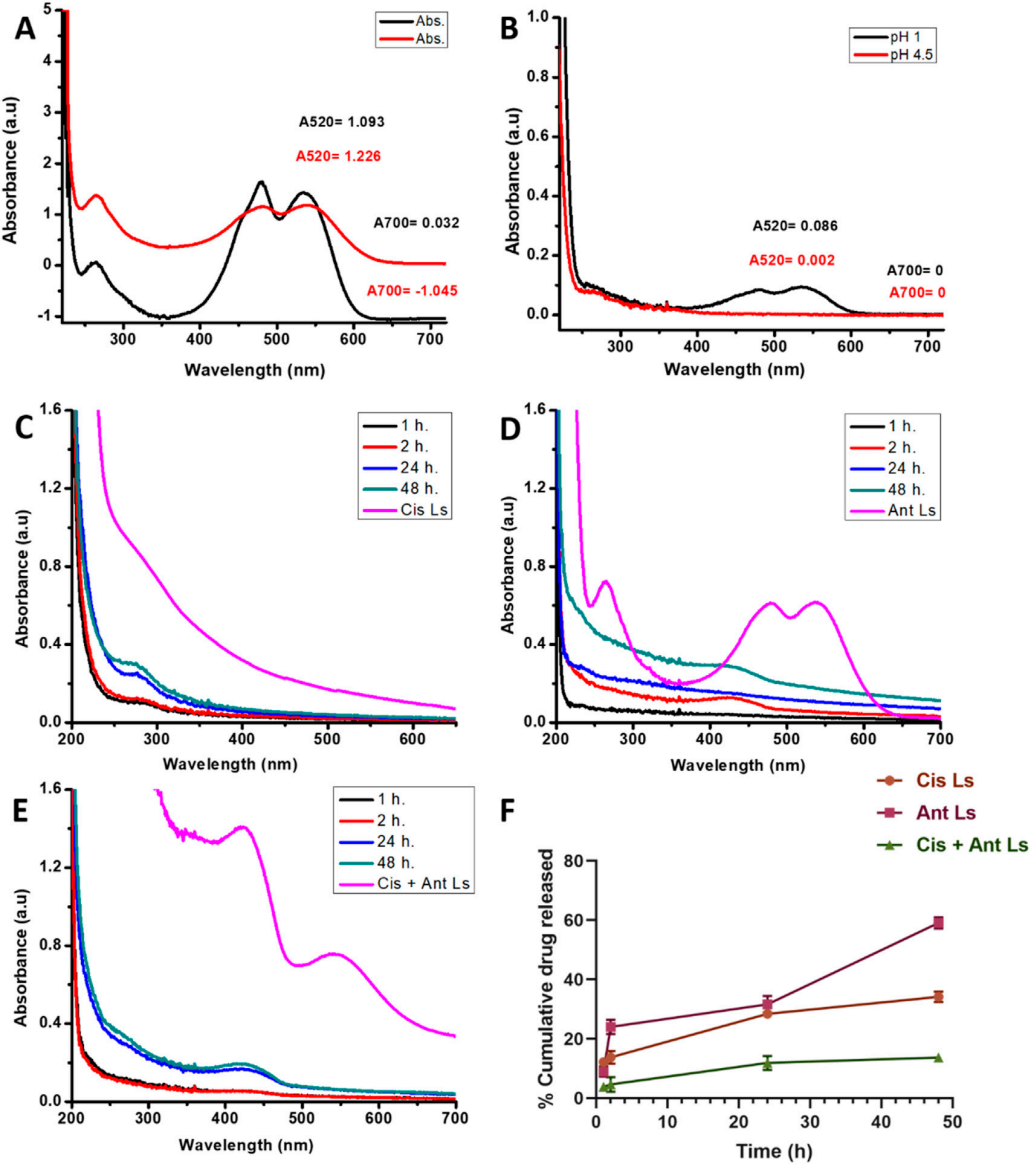
**(A)** Total Ant Quantification: Spectrophotometric analysis of beetroot extract using pH-differential method at pH 1.0 and pH 4.5. **(B)** Encapsulation efficiency from total Ant in the supernatant after encapsulation. **(C–F)** The *in vitro* release profile of liposomes for 48 h of Cis Ls **(C)**, Ant Ls **(D)**, Cis + Ant Ls **(E)**, and percentage of cumulative drug released **(F)**. Data was represented as mean ± SEM (n = 3).

The total Ant concentration in the beetroot extract was measured using the pH differential method. This recognized spectrophotometric approach depends on the structural alteration of anthocyanins at varying pH levels. Absorbance studies were conducted at 520 nm and 700 nm in two distinct buffer systems: pH 1.0 (potassium chloride buffer) and pH 4.5 (sodium acetate buffer). The adjusted absorbance values were computed as follows: at pH 1.0, (A520−A700) = 1.226−(−1.045) = 2.271 and at pH 4.5, (A520−A700) = 1.093–0.032 = 1.061 (Figure 3A). Total Ant content = $\frac{\left(2.271-1.061\right)\times 449.2\times 50\times 1000}{26,900\times 1}$ = 1.210 × 834.94 = 1,010.23 mg/L. The beetroot extract contains 1,010.23 mg/L of total Ant, indicating a substantial pigment content that aligns with its deep red hue.

### 3.3 Determination of Ant encapsulation efficiency (EE)

The EE of beetroot Ant was assessed by comparing the crude extract with the post-encapsulation supernatant using the pH differential technique. Preliminary spectrophotometric assessments at pH 1.0 and 4.5 produced corrected absorbance values of 0.086 and 0.002, respectively (Figure 3B), validating accurate anthocyanin characterization with enhanced stability in acidic environments. The examination of the supernatant indicated an unencapsulated Ant concentration of 70.13 mg/L, determined using cyanidin-3-glucoside as a reference (MW = 449.2 g/mol, ε = 26,900 L/mol cm) with a dilution factor of 50. The EE was then determined by comparing the encapsulated Ant content to the total starting content. EE (%) = (Encapsulated Ant/Total Ant) × 100. In contrast to the crude extract’s significant Ant concentration of 1,010.23 mg/L, the lower supernatant value of 940.10 mg/L (93.06%) signifies efficient encapsulation, demonstrating excellent incorporation of Ant into the matrix. These data indicate high EE, implying strong safeguarding of the bioactive chemicals.

### 3.4 *In vitro* release kinetics of Cis Ls and/or Ant Ls

**FIGURE 4:**
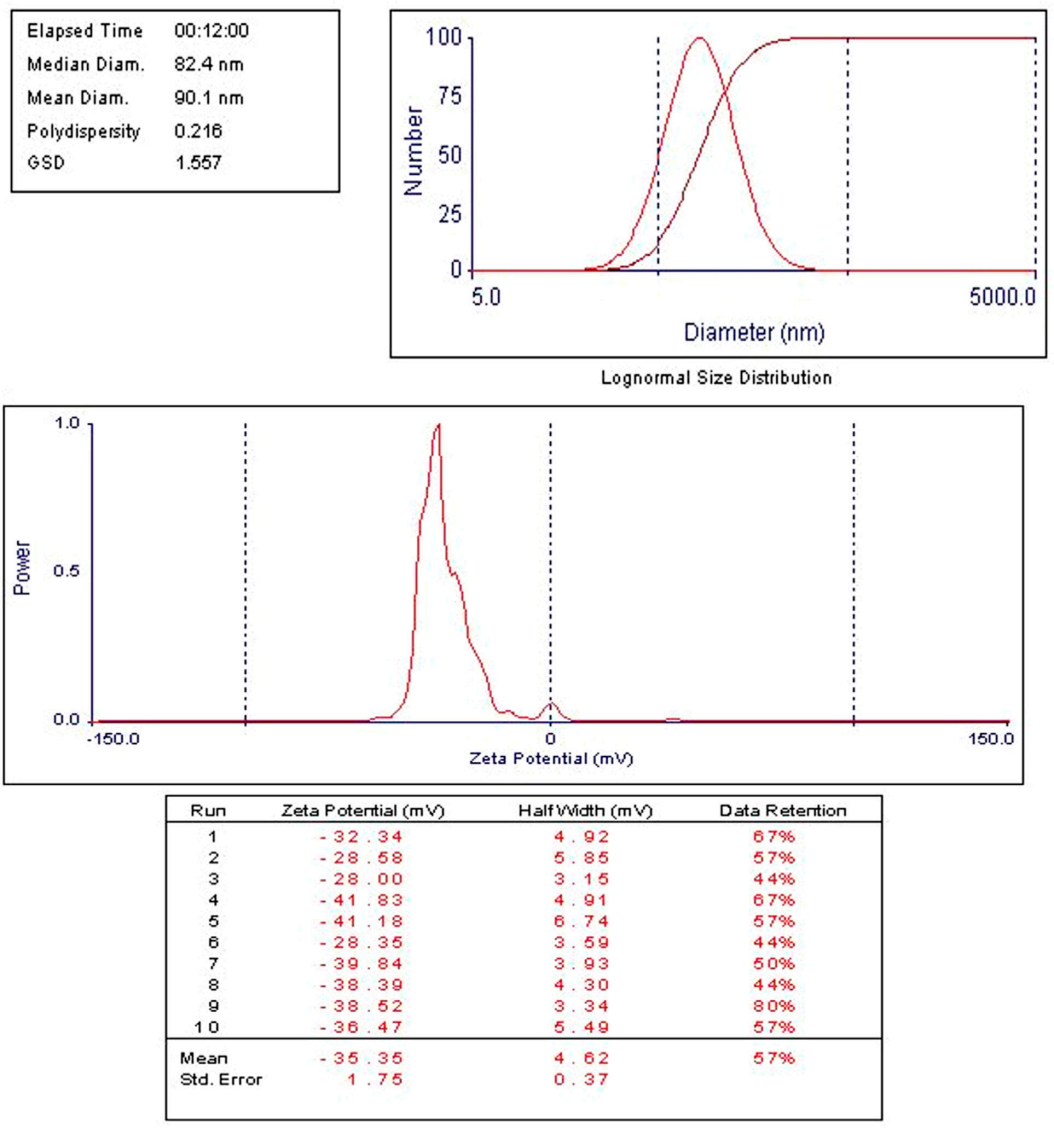
Zeta potential measured by dynamic light scattering (DLS) and nano-sizer for anthocyanin liposomes. Ant Ls demonstrated a high encapsulation efficiency of 93.06%.

The time-dependent release profiles of Cis, Ant, or Cis + Ant from different formulations demonstrated significant variations among treatment groups. The Cis Ls group exhibited a gradual release pattern, reaching 12.17% ± 1.35% at 1 h, 13.75% ± 2.12% at 2 h, and progressively increasing to 34.16% ± 1.74% by 48 h. In contrast, the Ant Ls group showed markedly enhanced release kinetics, with percentages escalating from 8.97% ± 1.65% at 1 h to 59.11% ± 1.87% at 48 h, representing the most substantial cumulative release among all groups. The combination group (Cis + Ant Ls) displayed intermediate release characteristics, maintaining lower but sustained release values throughout the study period (3.82% ± 1.21% at 1 h to 13.68% ± 1.30% at 48 h) (Figures 3C–F). Comparative analysis revealed that all liposomes achieved substantially high release rates. The current data further extends these observations, with the Ant Ls group showing superior release performance, reaching nearly 60% cumulative release by 48 h. Anthocyanin-loaded liposomes showed a mean size of 90 nm and a strong negative zeta potential (−35.35 ± 1.75 mV) as shown in Figure 4.

### 3.5 Impact of Ant Ls and/or Cis Ls on the viability of HCT 116 and vero cells

**FIGURE 5:**
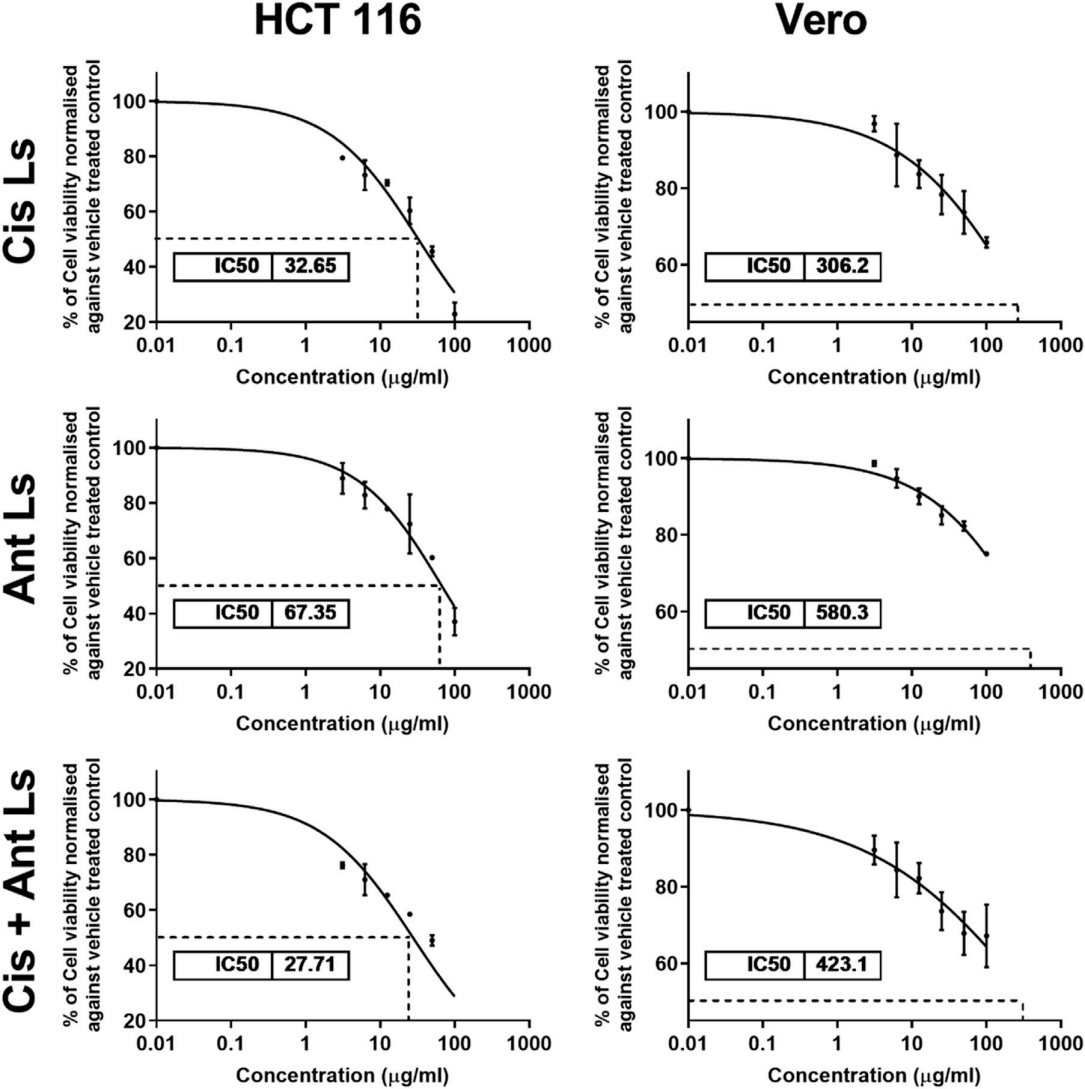
MTT assay results show the IC_50_ values of cisplatin liposomes (Cis Ls), anthocyanin liposomes (Ant Ls), and the combination of cisplatin and anthocyanin Ls on HCT 116 and Vero cell lines. Each data point represents the mean of three independent experiments (n = 3).

The MTT assay results demonstrated that Ant Ls exhibited a cytotoxic effect on HCT 116 cells, with IC_50_ of 67.35 ± 1.86 μg/mL, while Cis Ls showed slightly higher cytotoxicity, with IC_50_ of 32.65 ± 1.20 μg/mL. The cotreatment (Cis + Ant Ls) further reduced cell viability, yielding the lowest IC_50_ 27.71 ± 1.01 μg/mL in HCT 116 cells (Figure 5). Conversely, the Vero cells showed no significant (P > 0.05) cytotoxic effects when treated with Cis Ls, Ant Ls, or Cis + Ant Ls resulting in IC_50_ values of 306.20 ± 2.39, 580.30 ± 4.01, and 423.10 ± 3.25 μg/mL, respectively (Figure 5).

### 3.6 Influence of Ant Ls and/or Cis Ls on tumor volume and EAC cell count

**FIGURE 6:**
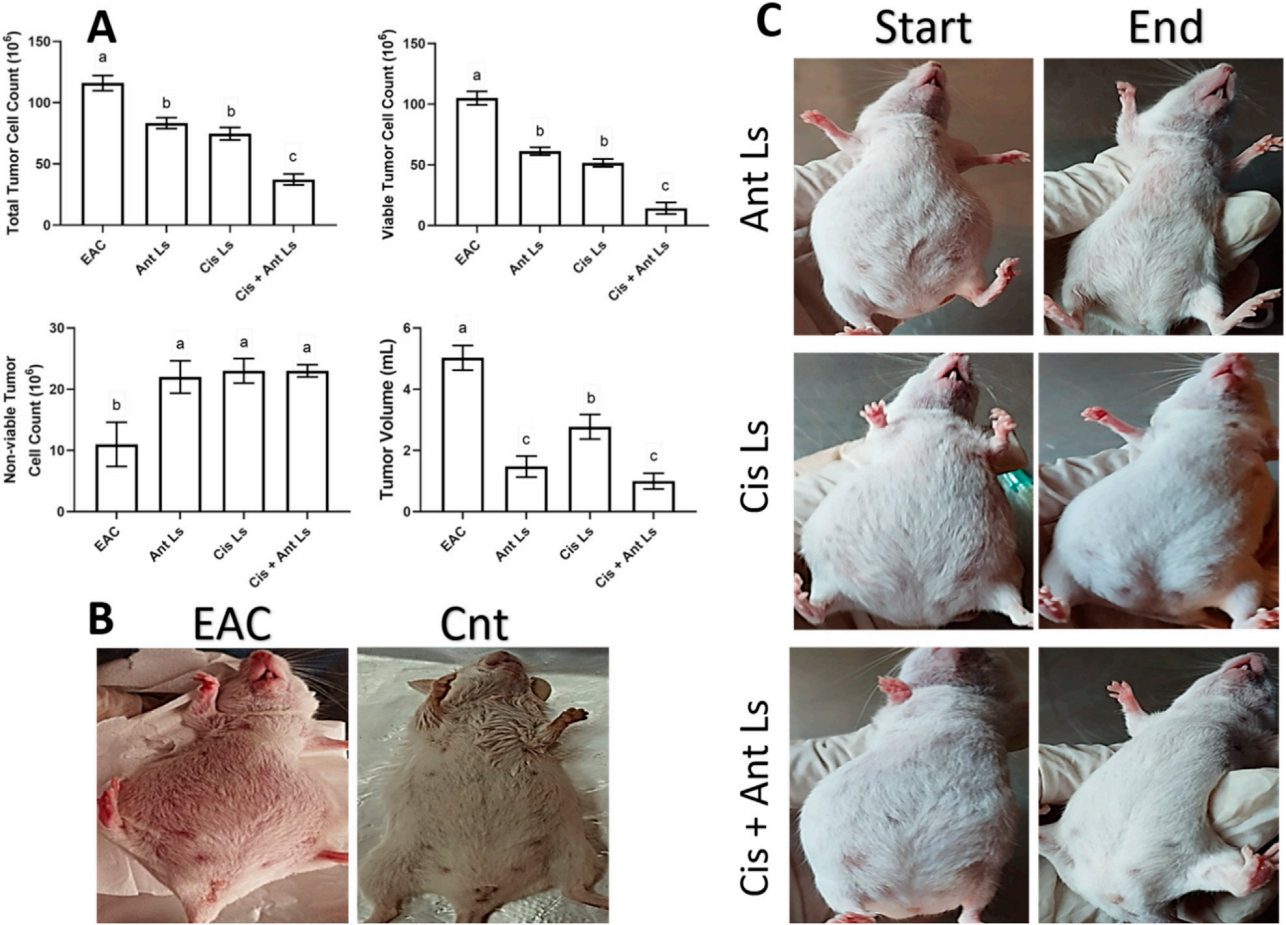
Effect of Ant Ls, Cis Ls, and Cis + Ant Ls treatments of EAC burden. **(A)** The effect of treatments on total tumor cell count, viable and non-viable tumor cell count, and tumor volume. Results were expressed as mean ± SEM (n = 7). Columns carrying different letters are significantly different [a (highest value) - c (lowest value)], denoting significant differences at P < 0.05. **(B)** Images of mice in the EAC and control (Cnt) group. **(C)** Images of mice in Ant Ls, Cis Ls, and Cis + Ant Ls groups, before and after treatment.

Compared to the untreated EAC group, all three treatment groups (Cis Ls, Ant Ls, and Cis + Ant Ls) showed a notable (P < 0.05) reduction in total tumor cell counts, viable tumor cells, and ascitic fluid volume, along with a significant (P < 0.05) increase in non-viable cells (Figure 6). Among the treated groups, the Cis + Ant Ls combination stood out as the most effective, with the lowest total and viable tumor cell counts and the smallest ascitic fluid volume. The Ant Ls group also showed strong suppression of tumor growth, though slightly less pronounced than the combination treatment, while the Cis Ls group had a more modest effect.

### 3.7 Impact of Ant Ls and/or Cis Ls on EAC cells morphology

**FIGURE 7:**
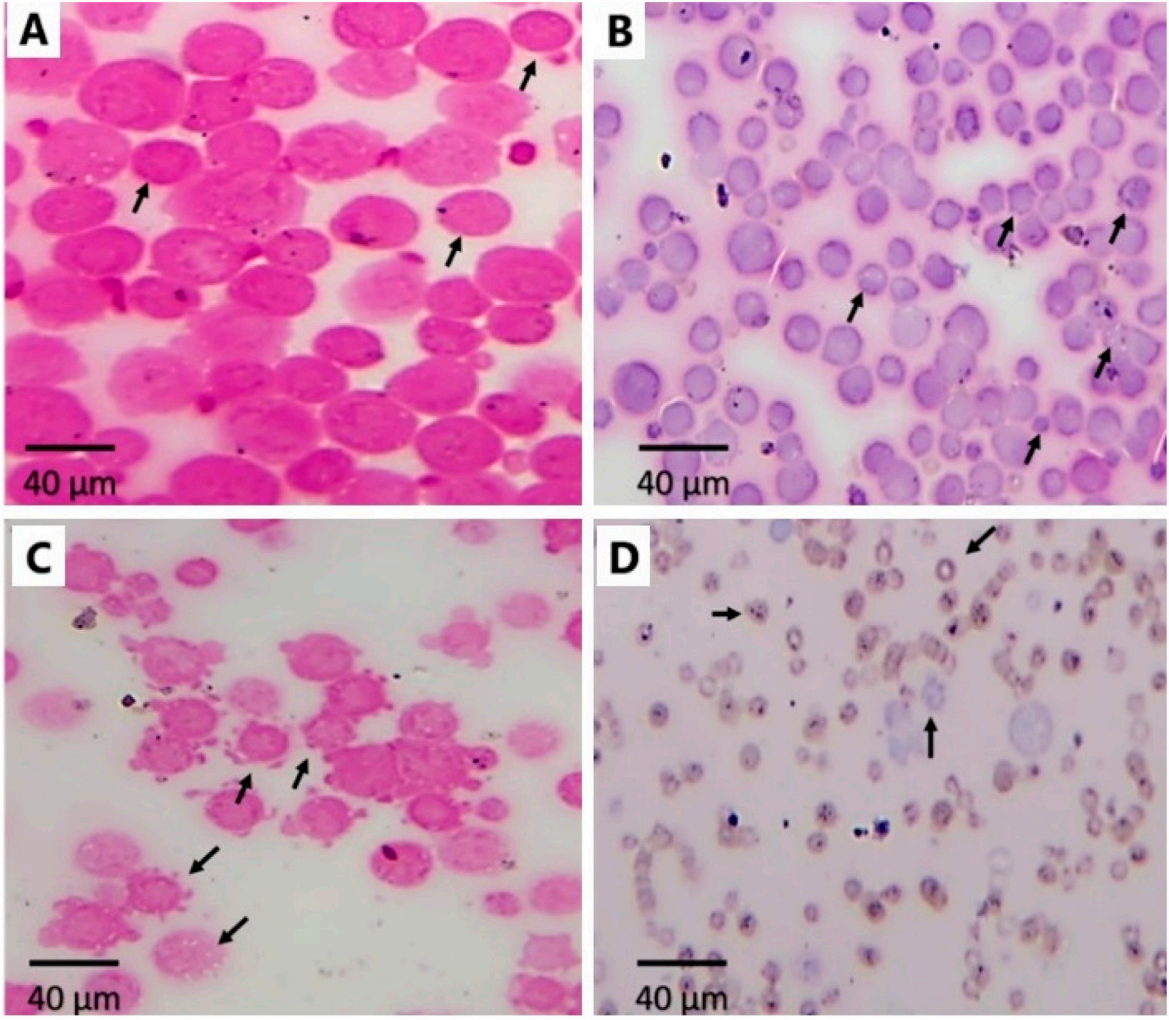
Effect of Ant Ls and/or Cis Ls treatments on EAC. Optical detection of apoptosis using H&E staining in untreated EAC cells **(A)**, EAC cells treated with Cis Ls **(B)**, EAC cells treated with Ant Ls **(C)**, and EAC cells treated with Cis + Ant Ls **(D)**. Arrows refer to normal round viable EAC cells **(A)** and apoptotic EAC **(B–D)**.

The untreated EAC cells were densely packed and round with intact membranes and homogenous cytoplasm (arrows, Figure 7A), indicating high cell viability and proliferation. EAC treated with Cis Ls displayed notable morphological changes: nuclear shrinkage, vacuoles formed, and membrane irregularities, indicative of apoptosis (arrows, Figure 7B). EAC treated with Ant Ls also showed disintegrated membranes, apoptotic bodies formed, cytoplasmic vacuolization, and other signs of necrosis and apoptosis (arrows, Figure 7C). EAC treated with Cis and Ant Ls demonstrated the most extensive cellular damage, including shrinkage, fragmented cells, and complete loss of structural integrity, reflecting enhanced synergistic cytotoxicity (arrows, Figure 7D).

### 3.8 Effect of Ant Ls and/or Cis Ls on liver and kidney function

The EAC-bearing mice exhibited significant hepatic and renal damage, as evidenced by markedly elevated serum ALT, AST, urea, and creatinine levels compared to healthy controls. All treatment regimens significantly (P < 0.05) mitigated hepatic and renal dysfunction. Ant Ls and the combination therapy (Cis + Ant Ls) demonstrated the most potent hepatoprotective effects, reducing ALT, AST, urea, and creatinine to near-normal levels. However, Cis Ls monotherapy provided a more modest improvement (Table 2).

**TABLE 2:** Liver and Kidney function parameters in EAC-bearing mice after treatment with Cis Ls, Ant Ls, and Cis + Ant Ls.

Group	ALT (U/L)	AST (U/L)	Urea (mg/dL)	Creatinine (mg/dL)
Cnt	38.41 ± 1.48^c^	40.15 ± 2.07^d^	26.23 ± 1.15^e^	0.94 ± 0.04^c^
EAC	99.52 ± 3.45^a^	113.49 ± 4.27^a^	75.98 ± 2.29^a^	1.96 ± 0.13^a^
EAC + Cis Ls	47.14 ± 1.96^b^	66.34 ± 2.81^b^	62.75 ± 1.99^b^	1.67 ± 0.09^b^
EAC + Ant Ls	39.17 ± 1.54^c^	45.39 ± 2.18^c,d^	53.42 ± 1.76^c^	1.14 ± 0.04^c^
EAC + Cis + Ant Ls	41.90 ± 1.86^c^	50.63 ± 2.65^c^	38.92 ± 1.36^d^	1.21 ± 0.05^c^

Data are presented as mean ± SEM (n = 7/group). Columns carrying different letters [a (the highest value)–e (the lowest value)] are significantly different at P < 0.05. All groups were compared to control group. Cnt, control group; EAC, ehrlich ascites carcinoma group; cisplatin liposomes (Cis Ls), anthocyanin liposomes (Ant Ls), and the combination of cisplatin and anthocyanin Ls (Cis + Ant Ls).

### 3.9 Influence of Ant Ls and/or Cis Ls on apoptosis and antioxidant genes in EAC cells

**FIGURE 8:**
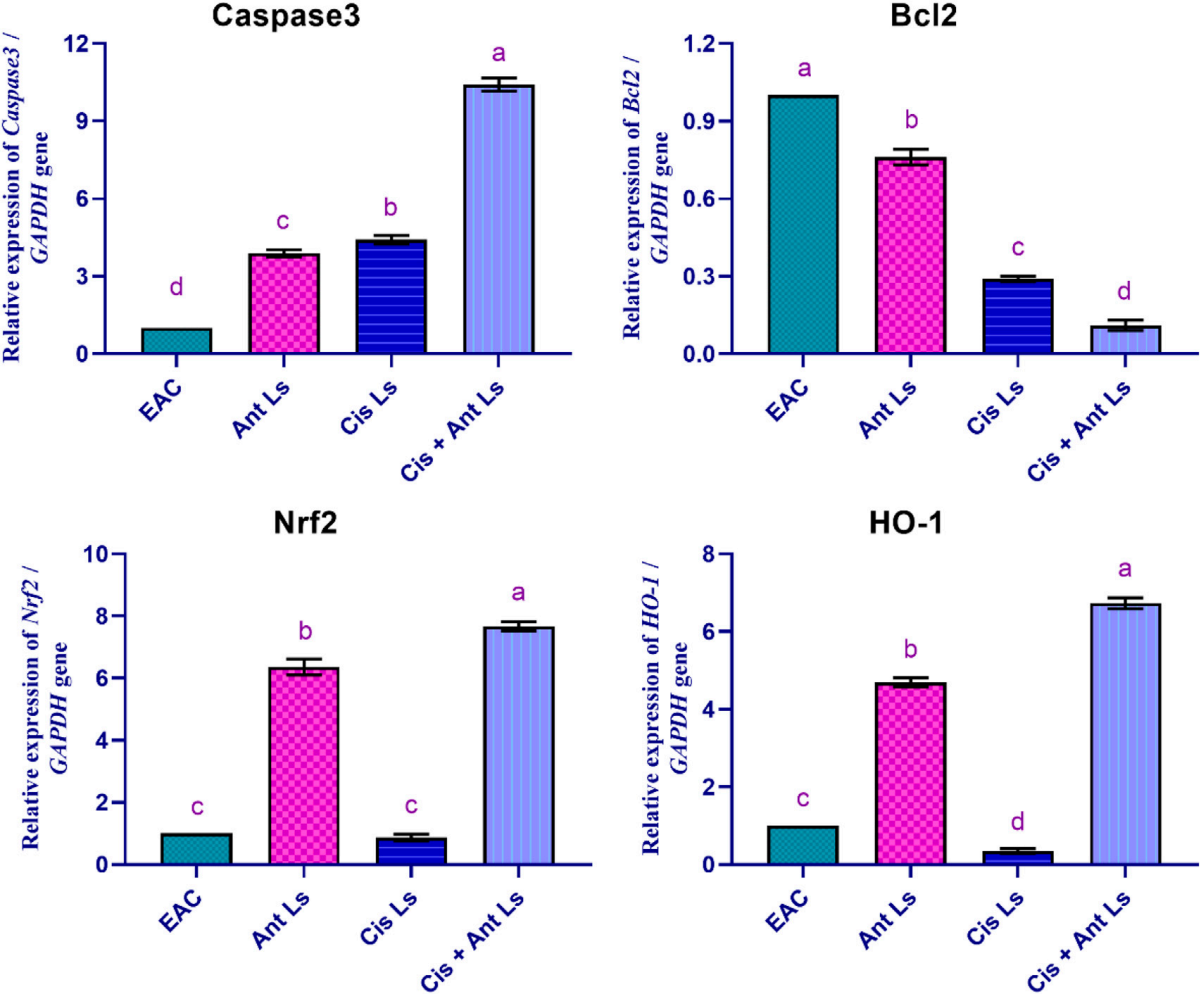
Effect of Ant Ls, Cis Ls, and Cis + Ant Ls treatments on relative mRNA expressions of Caspase3, Bcl2, Nrf2, and HO-1 in EAC cells. Values are given as mean ± SEM (n = 3/group). Various letters [a (highest value) - d (lowest value)] denote significant differences at P < 0.05. All groups were compared to each other.

The results indicated that Cis + Ant Ls is the most effective treatment in increasing apoptotic markers Caspase3 (10.41 ± 0.25-fold change) while reducing the anti-apoptotic marker *Bcl2* (0.11 ± 0.02-fold change) in EAC cells compared with Cis Ls and Ant Ls (Caspase3, 4.41 ± 0.17 and 3.89 ± 0.14-fold change, and *Bcl2*, 0.29 ± 0.01 and 0.76 ± 0.03-fold change, respectively) (Figure 8). Moreover, the EAC group exhibited minimal *Nrf2* expression (0.70 ± 0.06-fold change) in the EAC cells. Ant Ls substantially enhanced *Nrf2* expression (6.36 ± 0.26-fold change). Like the control, Cis Ls exhibited low *Nrf2* expression (0.87 ± 0.11-fold change), while Cis + Ant Ls demonstrated the most significant (P < 0.05) *Nrf2* expression (7.67 ± 0.15-fold change). Cis Ls reduced *HO-1* expression (0.35 ± 0.06-fold change); Ant Ls substantially enhanced *HO-1* expression (4.69 ± 0.12-fold change); Cis + Ant Ls significantly (P < 0.05) maximized this expression (6.73 ± 0.14-fold change) (Figure 8).

### 3.10 Effect of Ant Ls and/or Cis Ls on VEGF, IL1β, and MMP9 genes in EAC cells

**FIGURE 9:**
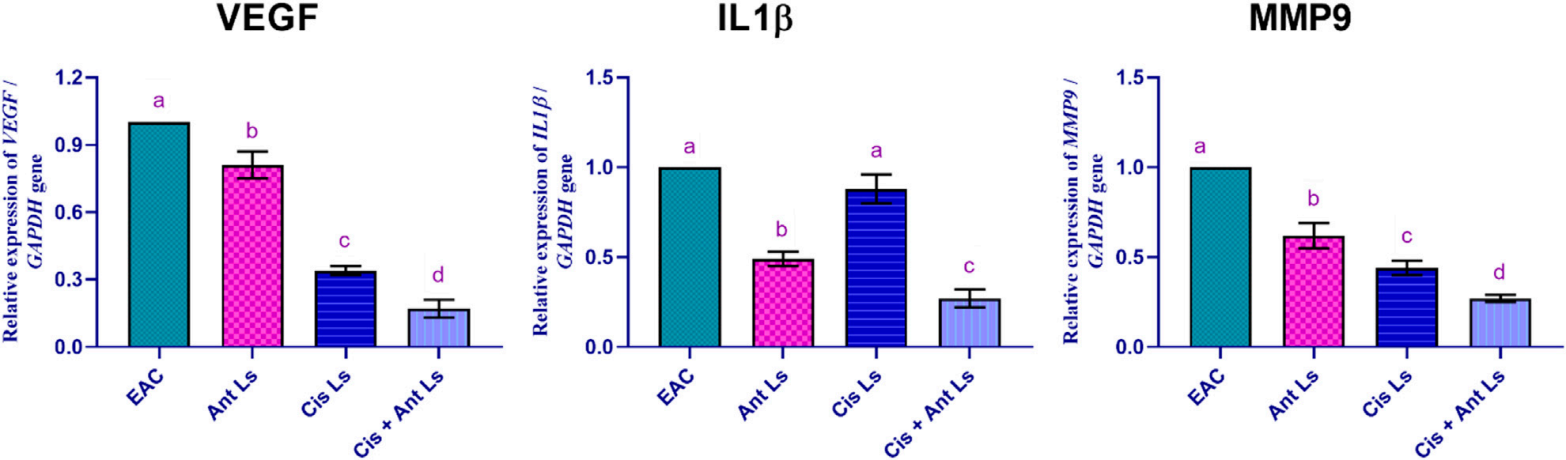
Effect of Ant Ls, Cis Ls, and Cis + Ant Ls treatments on relative mRNA expression of VEGF, IL1β, and MMP9 in EAC cells. Values are given as mean ± SEM (n = 3/group). Various letters [a (highest value) - d (lowest value)] denote significant differences at P < 0.05. All groups were compared to each other.

The EAC group showed the highest *VEGF* expression (1.00 ± 0.00-fold change). Ant Ls modestly reduced *VEGF* expression (0.81 ± 0.06-fold change). The Cis Ls group showed a more substantial decrease in *VEGF* levels (0.34 ± 0.02-fold change) than the EAC group. Additionally, Cis + Ant Ls showed the lowest *VEGF* expression (0.17 ± 0.04-fold change), indicating a combined, strong suppression of angiogenesis, effectively limiting the tumor’s capacity for growth and spread (Figure 9).

Moreover, the EAC group exhibited the greatest *IL1β* expression level (1.00 ± 0.00-fold change). Ant Ls reduced *IL1β* expression (0.49 ± 0.04-fold change) compared to the EAC. Cis Ls diminished *IL1β* levels (0.88 ± 0.08-fold change). The combination of Cis and Ant Ls resulted in the minimal production of *IL1β* (0.27 ± 0.05-fold change), suggesting a possible anti-inflammatory action that may hinder tumor development and spread.

The EAC group demonstrated the most significant (P < 0.05) levels of *MMP9* expression (1.00 ± 0.00-fold change). Ant Ls exhibited a modest reduction in *MMP9* expression (0.62 ± 0.07-fold change). Cis Ls further reduced *MMP9* levels (0.44 ± 0.04-fold change). Cis and Ant Ls demonstrated the lowest expression of *MMP9* (0.27 ± 0.02-fold change), indicating a significant (P < 0.05) synergistic impact in inhibiting tumor cell migration and metastasis (Figure 9).

### 3.11 Influence of Cis Ls and/or Ant Ls treatments on liver histopathology

**FIGURE 10:**
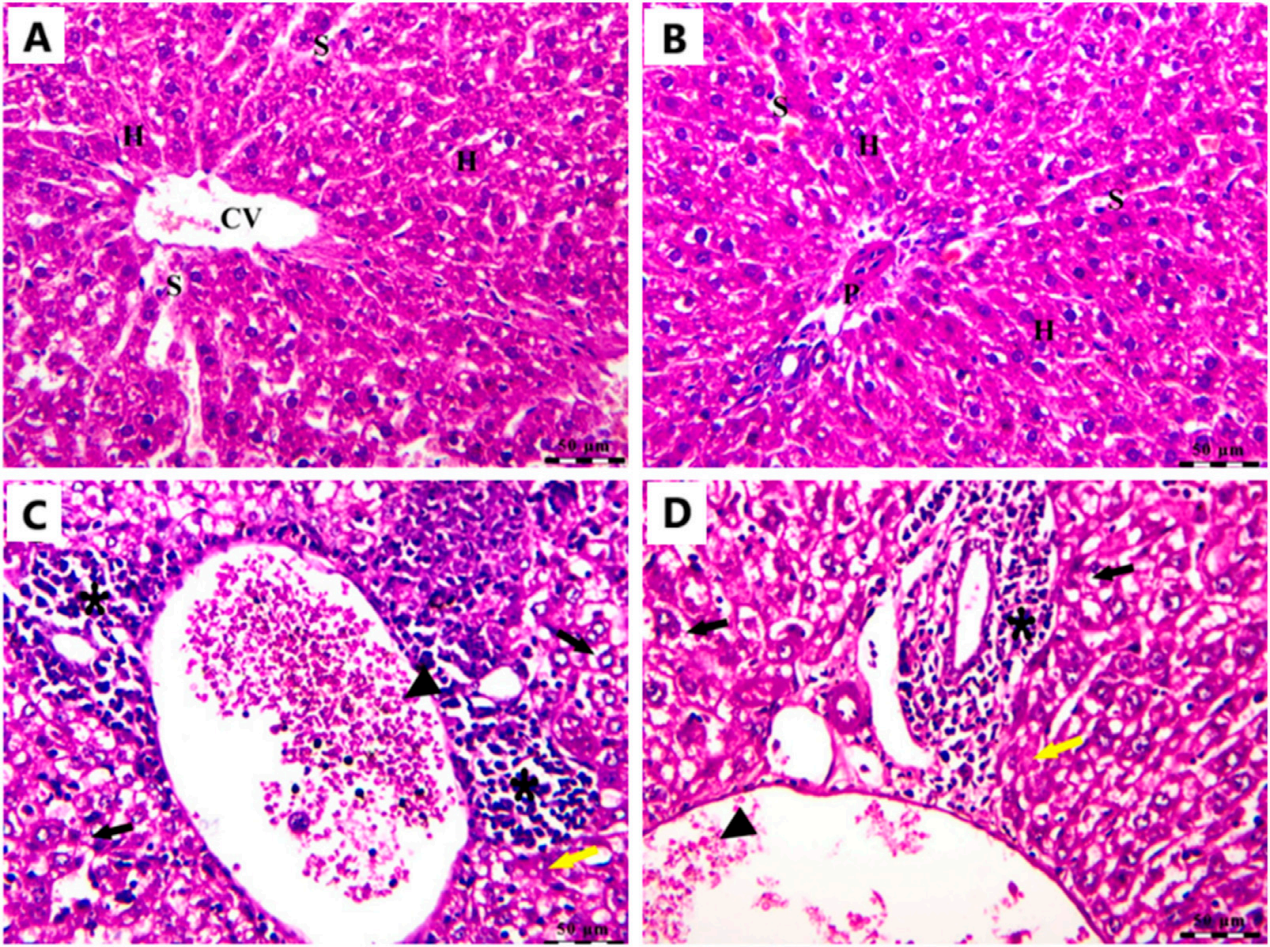
Photomicrograph of the liver stained by H&E in the Cnt group **(A,B)** and the EAC group **(C,D)**. All labels are indicated in the main text. Scale bars = 50 µm.

**FIGURE 11:**
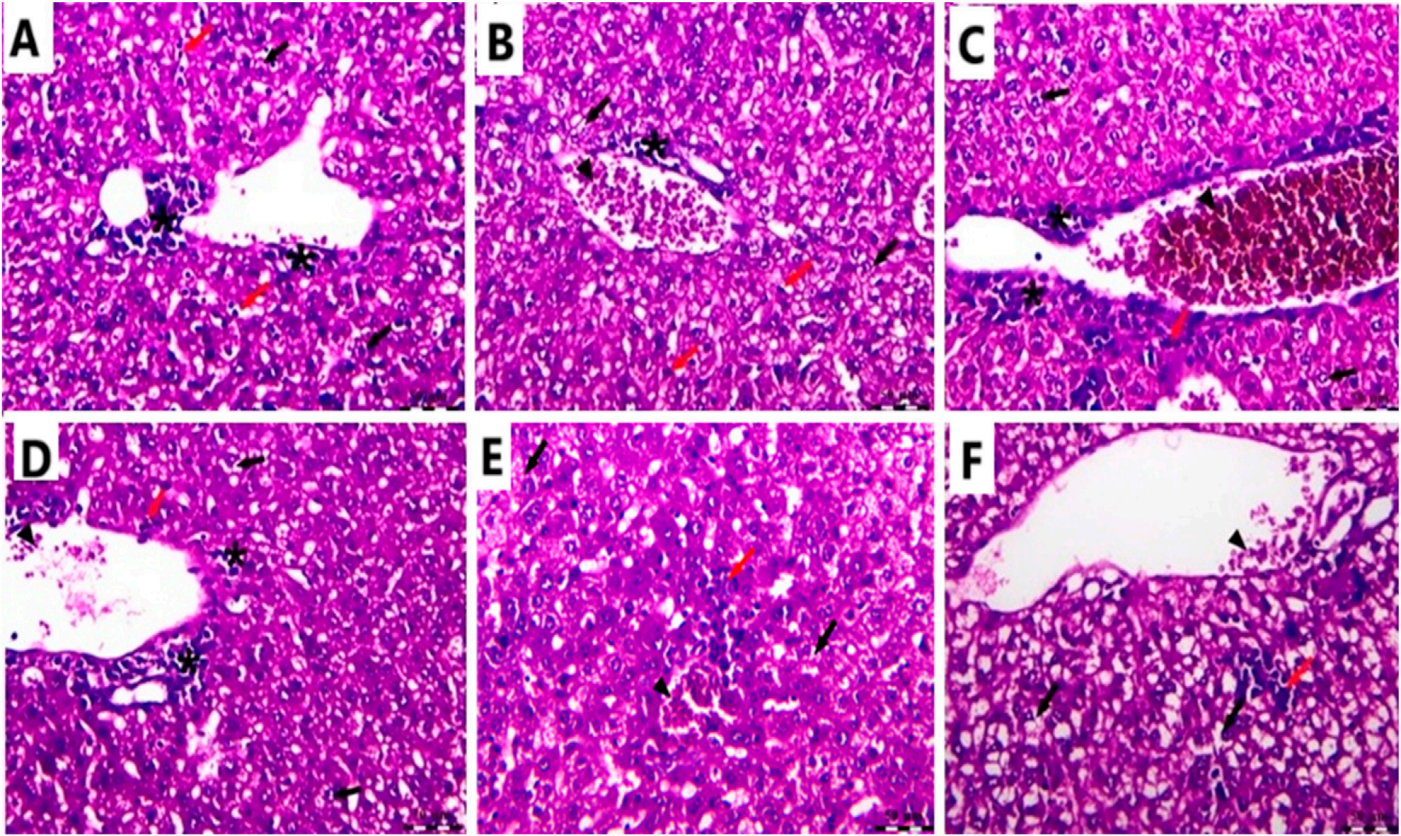
Photomicrograph of the liver stained by H&E in the Cis LS group **(A,B)**; the Ant Ls group **(C,D)**; and the Cis + Ant Ls group **(E,F)**. All labels are indicated in the main text. Scale bars = 50 µm.

In the Cnt group, the liver has a healthy structure with polyhedral-shaped hepatocytes arranged in a cord-like pattern (H) radiating from the intact central vein (CV) and separated by blood sinusoids (S) and an intact portal artery (P) (Figures 10A,B). In the EAC group, notable pathological alterations are evident, including aggregations of polymorphic neoplastic cells (asterisks) around the congested central vein (arrowheads), swelling and vacuolar degeneration of hepatocytes (black arrows), and an area of hepatocellular necrosis (yellow arrows) (Figures 10C,D). Cis Ls treatment decreased neoplastic cell clusters (asterisks) around the congested hepatic blood vessels (arrowheads). However, isolated polymorphic neoplastic cells remained (red arrows), and some hepatocytes exhibited swelling and vacuolar degeneration (black arrows) (Figures 11A,B). Ant Ls therapy has a therapeutic benefit by diminishing neoplastic cell clusters (asterisks) around the mildly congested hepatic blood vessels (arrowheads). Still, some hepatocyte degradation persists (black arrows) in addition to the number of sporadic polymorphic neoplastic cells (red arrow) (Figures 11C,D). Cis + Ant Ls has the most pronounced ameliorative benefits, shown by diminished clusters of degenerative neoplastic cells (red arrows), decreased congestion of hepatic blood vessels (arrowheads), and reduced hepatocellular degeneration symptoms (black arrows) (Figures 11E,F). The findings indicate that Cis and Ant, alone and in conjunction, may provide therapeutic advantages in alleviating liver damage caused by migratory EAC cells, with their combination yielding superior protective effects.

### 3.12 Effects of Cis Ls and/or Ant Ls on kidney histopathology

**FIGURE 12:**
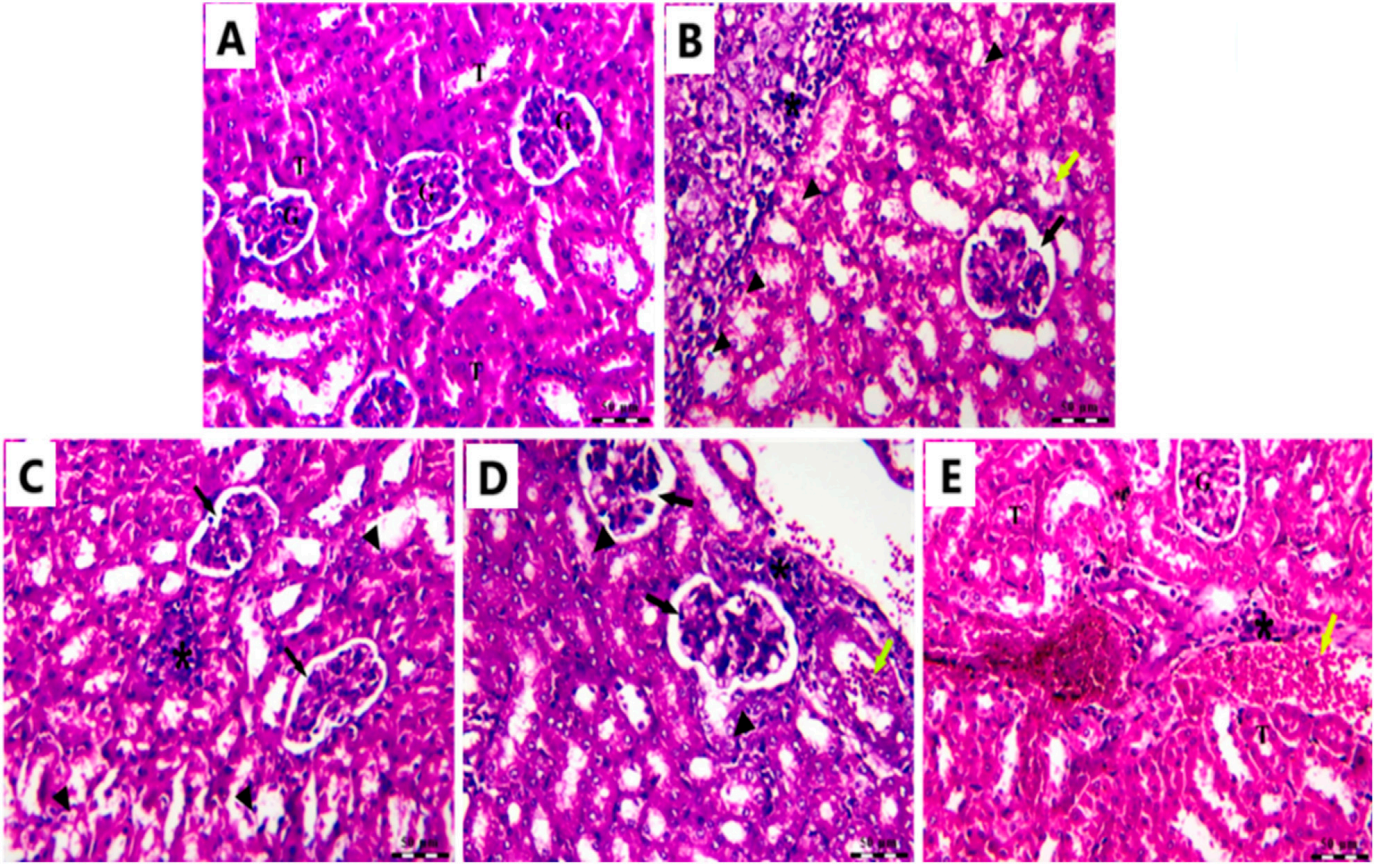
Photomicrograph of the kidney stained by H&E in the Cnt group **(A)**, EAC group **(B)**, Ant Ls group **(C)**, Cis Ls group **(D)**, and Cis + Ant Ls group **(E)**.

In the Cnt group, the kidney had a robust architecture, featuring intact glomeruli (G) encased by Bowman’s capsules and well-structured proximal and distal convoluted tubules (T) (Figure 12A). In the (EAC) group, pathological alterations are observed, including clusters of polymorphic neoplastic cells (asterisk), degeneration of tubular epithelium (arrowheads), hyaline casts (yellow arrow), atrophied glomeruli, and enlarged capsular space (black arrow) (Figure 12B). Individual treatment with Cis Ls or Ant Ls demonstrated modest enhancement, characterized by reduced clusters of degenerated neoplastic cells (asterisk) around congested renal blood vessels (yellow arrow), accompanied by minimal glomerular shrinkage (black arrow). However, degeneration of the tubular epithelium continues (arrowheads) (Figures 12C,D). The Cis + Ant Ls group had the most pronounced ameliorative impact, characterized by minimal clusters of degenerated neoplastic cells (asterisk), diminished vascular congestion (yellow arrow), intact tubular epithelium (T), and retained glomeruli (G) (Figure 12E). Our data demonstrate that Cis and Ant, either alone or in combination, may help mitigate EAC-induced kidney injury, with the combined treatment offering the most robust protection.

## 4 Discussion

Whether administered alone or in conjunction with other anticancer drugs, Cis is a frequently prescribed chemotherapy. However, its use is restricted due to the potential serious side effects. Cis NPs, which boost therapy efficacy while reducing side effects, were created to circumvent these constraints (Awad et al., 2024b; Joshy et al., 2022). Cis Ls compositions target cancer cells, increase Cis bioavailability at the tumor site, reduce systemic toxicity, and boost EAC therapy efficacy (Ledezma-Gallegos et al., 2020). Although Ants have shown promise in inhibiting cancer progression, their poor bioavailability and solubility have limited their use (Awad et al., 2024a; Karthikeyan et al., 2020; Shen et al., 2022). Therefore, Ant Ls formulations aim to overcome these constraints by enhancing the stability and bioavailability of Ants through their encapsulation in Ls. This study aimed to determine whether encapsulated Ant and Cis Ls coated with chitosan would effectively treat EAC cells. This is the first study to indicate that combined therapy with Ant and Cis Ls can successfully target EAC cells by inducing apoptosis, increasing antioxidant capacity, and inhibiting inflammation, metastasis, and angiogenesis.

FTIR analysis confirmed the preservation of the chemical structure of Cis and Ant in their nanoparticle formulations. The presence of specific peaks corresponding to platinum and ammonium bonds in Cis and Cis Ls (565 cm^−1^ and 466 cm^−1^) and tensional trembles of ammonium groups at ∼3,300 cm^−1^ and 3,400 cm^−1^ aligns with previous research, indicating structural stability (Awad et al., 2024b; Babaei et al., 2019). Similarly, the Ant FTIR spectrum revealed characteristic–OH stretching vibrations (3,266 cm^−1^ in Ant and 3,277 cm^−1^ in Ant Ls) and C=N stretching bands at 1,645 cm^−1^ and 1,558 cm^−1^, which are consistent with prior findings (Jeyaram and Geethakrishnan, 2020). The successful encapsulation and preservation of functional groups in liposomal formulations suggest their potential for effective drug delivery. TEM analysis further confirmed the uniformity and spherical shape of the liposomes, with diameters ranging from 70 to 130 nm. This size range optimizes enhanced bioavailability and targeted drug delivery, ensuring better retention and cellular uptake (Allen and Cullis, 2013). Ant Ls demonstrated a high encapsulation efficiency of 93.06% and exhibited sustained release profiles, achieving the highest cumulative drug release of 59.11% at 48 h. These results confirm the enhanced delivery capability of the encapsulated systems, with the Cis + Ant Ls group formulation emerging as the most effective in promoting drug release over extended periods. The sustained release profiles observed across all liposome formulations suggest their potential for prolonged therapeutic delivery applications and validate their suitability for further *in vivo* therapeutic evaluation.

The lower cumulative release observed for Cis + Ant Ls likely reflects altered intraliposomal milieu and strengthened drug–bilayer interactions during co-encapsulation. Co-loaded systems can display reduced membrane permeability due to competitive partitioning, ion-pairing/complexation, and increased bilayer order, thereby attenuating burst release and slowing diffusion. These effects have been reported for co-loaded liposomes where drug–drug and drug–lipid interactions modulate release kinetics. We interpret the higher Ant Ls release as greater membrane permeability/partitioning of Ant in this lipid matrix, yielding sustained but appreciable drug availability *in vitro*. Conversely, the reduced release from Cis + Ant Ls likely arises from co-loading effects that strengthen cargo retention; such retention may benefit circulation stability.

*In vitro* studies revealed selective cytotoxicity, showing potent anticancer effects against colon cancer cells while maintaining safety in normal cells. Cis Ls and/or Ant Ls demonstrated significant (P < 0.05) cytotoxic effects on HCT 116 colon cancer cells while exhibiting minimal toxicity to normal Vero cells. These results suggest combining Cis and Ant in one liposome may enhance antitumor activity of Cis and may decrease Cis toxicity. The anticancer potentials of these liposomal formulations were also verified on another cancer type, the EAC. Pathological analysis of EAC cells provides valuable insights into the effects of Cis Ls and/or Ant Ls treatments on cellular architecture and tumor progression. In the untreated EAC control group, tumor cells were characterized by high mitotic activity, pleomorphic nuclei, and a dense cellular architecture indicative of rapid proliferation and malignancy. However, treated cells showed necrosis and apoptosis, including disintegrated membranes, apoptotic bodies formed, and cytoplasmic vacuolization. Furthermore, Cis Ls and/or Ant Ls treatments caused decreases in total tumor cell counts, viable tumor cells, and ascitic fluid volume and increases in non-viable cells, indicating a decrease in tumor burden. EAC cells treated with Cis Ls and/or Ant Ls also showed a significant (P ˂ 0.05) drop in *Bcl2* expression and a rise in Caspase 3. This finding confirm that the induced cell death could be dependent on apoptosis. In support, previous studied reported that Ants can increase the anticancer efficacy of Cis by affecting significant (P < 0.05) signaling pathways related to death (Kowalczyk et al., 2024; Paramanantham et al., 2020). Awad et al., (2024a), Awad et al. (2024b) claim that Cis and Ants trapped in carbon nanotubes have a synergistic impact and increase HepG2 and MCF7 cancer cell killing. Li et al. (2014) found that Ants and their metabolites can induce apoptosis in cancer cells. Cis-NPs and Ls were also used as a targeted treatment for cancer cells (Li et al., 2012; Yang et al., 2017) and boosted EAC therapy efficacy (Ledezma-Gallegos et al., 2020).

The observed 10.4-fold upregulation of caspase-3 in the Cis + Ant Ls group suggests a strong pro-apoptotic response. Previous studies using liposomal Cis or plant-derived polyphenols typically report caspase-3 activation ranging between 2- to 8-fold in similar murine tumor models (Awad et al., 2024b; Elbialy and Mady, 2015) Thus, our result demonstrates a relatively higher induction, which may reflect the synergistic impact of co-encapsulating Ant with Cis. However, more mechanistic studies, including protein-level validation and time-course analysis, are warranted to confirm this effect. Interestingly, Ant Ls alone demonstrated a more pronounced effect on certain biological endpoints (e.g., apoptotic gene expression and reduction in viable tumor cell count) compared to Cis Ls or even the combination treatment. This observation may be attributed to several factors. First, Ants possess multifaceted bioactivity, including antioxidant, anti-inflammatory, and pro-apoptotic properties, which can contribute to broad-spectrum tumor suppression. Second, Ants are known to modulate multiple signaling pathways simultaneously, including caspase activation, Bcl-2 downregulation, and NF-κB suppression, potentially resulting in cumulative anticancer effects even at higher doses (Carvalho et al., 2017). In contrast, Cis’s action is more mechanistically narrow, primarily targeting DNA crosslinking, and its effectiveness can be limited by resistance mechanisms and toxicity. Additionally, the higher dose of Ant (100 mg/kg) used in the monotherapy group compared to 50 mg/kg in the combination may also account for the increased activity.

Using Cis has a main disadvantage in terms of a lack of specificity. It generates oxidative stress and blocks the activities of antioxidant enzymes, killing cancer cells and damaging healthy ones (Abass et al., 2024; Awad et al., 2024a; Awad et al., 2024b). In line with this idea, we found that EAC cells treated with Cis Ls significantly reduced the expression of the antioxidant *Nrf2* and *HO-1* genes. Conversely, the two antioxidant genes were significantly upregulated when EAC cells were co-treated with Cis and Ant Ls. This suggests the combined treatment promotes apoptosis and enhances the cellular antioxidant defense, potentially providing a dual therapeutic effect. This combination treatment could be promising for enhancing tumor cell death while protecting against oxidative stress. In support, Awad et al., (2024a), Awad et al. (2024b) also found that HepG2 and MCF7 cells have *Nrf2* and *HO-1* expression levels that were considerably increased by Ants NPs alone or in combination with Cis NPs compared to Cis NPs alone.

Metastasis and multi-organ failure are the leading causes of death from cancer. In contrast to treatment with Cis Ls alone, our research showed that treatment with Ant Ls alone or in combination with Cis Ls effectively reduced cancer cell migration and metastasis, as revealed by a considerable decrease in the metastasis-involved gene *MMP9* in EAC cells and decreased migratory EAC cells to the liver and kidney. Similarly, HepG2 and MCF7 treated with Ant NPs alone or in combination with Cis NPs using carbon nanotubes as carriers exhibited downregulated expression of *MMP9* (Awad et al., 2024a; Awad et al., 2024b). It has also been found that Ant-derived from black rice, can inhibit the migration and metastasis of various colon, breast, and liver cancer cells by targeting the same molecule (Chen et al., 2015; Shin et al., 2011).

According to the available evidence, effective cancer treatments possess anti-inflammatory and anti-angiogenic characteristics (Abdelwahab et al., 2019; Azaam et al., 2018; Othman et al., 2021). The inhibition of angiogenesis was indicated by the lowest *VEGF* expression in EAC cells treated with Cis Ls and/or Ant Ls. Among the treated groups, the combination therapy showed the greatest reduction in *IL1β* expression, suggesting a potential anti-inflammatory effect; cells treated with Ant Ls came second. At the same time, Cis Ls showed higher *IL1β* expression close to the untreated EAC cells. In agreement, it is well known that Cis can have detrimental effects cancer cells and disturb healthy ones at the same time (Domingo et al., 2022). Consistent with our findings, earlier research showed comparable anti-inflammatory and anti-angiogenic potentials for Cis + Ant NPs-loaded carbon nanotubes on HepG2 and MCF7 cells (Awad et al., 2024a; Awad et al., 2024b). Previous studies indicate Ants’ ability to reduce IL1β, TNFα, and VEGF levels, explaining their anticancer properties (Lin et al., 2017; Pan et al., 2010). Ants reduce TNFα and NFκB, which causes their fatal effect on some cancer cells (Venancio et al., 2017). By affecting the NF-κB pathway, Ants increase Cis’s anticancer potency (Kowalczyk et al., 2024; Paramanantham et al., 2020).

Mice carrying EAC showed notable liver and kidney damage, indicated by significantly (P < 0.05) higher levels of serum ALT, AST, urea, and creatinine compared to healthy mice. EAC cells can migrate from the peritoneal cavity to liver and kidney causing tissue damage (Tawfic et al., 2024b). All treatment approaches effectively reduced hepatic and renal impairment. Ant Ls alone and the combined therapy (Cis + Ant Ls) exhibited the strongest liver and kidney-protective effects, bringing ALT, AST, urea, and creatinine levels close to normal. In contrast, treatment with Cis Ls alone resulted in only a moderate recovery. Since Cis is known to cause liver and kidney damage, the observed reduction in serum ALT, AST, urea, and creatinine levels may be due to fewer EAC cells migrating to these organs following Cis Ls treatment, as supported by histopathological findings.

This study has certain constraints. First, EAC cells, which might not entirely reflect the complexity of actual tumors, were used *in vitro* and *in vivo*. More clinical research is required to confirm these conclusions in a larger sample size study on animals. Second, more studies will still be done on higher species’ long-term stability, biodistribution, and possible toxicity of chitosan-coated liposomal formulations (Cis Ls and Ant Ls). Third, although EAC is widespread, the effects of Cis on the rest of the body have not been investigated. It should be clarified that while the combination was found to be effective in the EAC model, these results cannot be generalized to other cancer types. Fourth, the encapsulation efficiency of Cis, as well as the co-encapsulation efficiency of Cis + Ant, was not experimentally determined due to methodological and resource constraints; future studies are warranted to address this aspect. Fifth, drug release was only evaluated at physiological pH (7.4) and not under acidic tumor-like conditions (pH 5.5–6.5), which should be addressed in future work to better reflect tumor-relevant environments. Finally, even if the combo therapy showed synergistic results, the molecular processes behind these interactions need more research.

## 5 Conclusion

This study successfully developed a novel liposomal co-delivery system combining cisplatin and anthocyanin (Cis + Ant Ls) for targeted cancer therapy. The optimized formulations demonstrated excellent drug encapsulation and sustained release properties. The treatment exhibited multiple therapeutic mechanisms, including the induction of apoptosis, antioxidant and anti-inflammatory effects, as well as the inhibition of angiogenesis and metastasis. This dual-action formulation represents a promising advancement in cancer therapy, combining chemotherapy with natural compounds in a targeted delivery system to improve both effectiveness and safety. The results support further development of this approach for clinical applications in oncology.

## Data Availability

The original contributions presented in the study are included in the article/supplementary material, further inquiries can be directed to the corresponding authors.

## Abbreviations

Ant, Anthocyanin; Bcl2, B cell lymphoma 2; Cis, Cisplatin; EAC, Ehrlich ascites carcinoma; FA, Folic acid; FTIR, Fourier Transform Infrared Spectrometer; H&E, Hematoxylin and eosin; Ls, Liposomes; MMP9, Matrix metalloproteinase 9; NPs, Nanoparticles; Nrf2, Nuclear factor erythroid 2-related factor 2; HO-1, Heme oxygenase-1; VEGF, Vascular endothelial growth factor.
